# A new LMS algorithm for analysis of atrial fibrillation signals

**DOI:** 10.1186/1475-925X-11-15

**Published:** 2012-03-26

**Authors:** Edward J Ciaccio, Angelo B Biviano, William Whang, Hasan Garan

**Affiliations:** 1 Department of Medicine – Division of Cardiology, Columbia University Medical Center, New York, USA; 2 Columbia University, Harkness Pavilion 804, 180 Fort Washington Avenue, New York, NY, 10032, USA

**Keywords:** Atrial fibrillation, Electrocardiogram, F wave, Fractionation, LMS algorithm, Mean-squared error

## Abstract

**Background:**

A biomedical signal can be defined by its extrinsic features (x-axis and y-axis shift and scale) and intrinsic features (shape after normalization of extrinsic features). In this study, an LMS algorithm utilizing the method of differential steepest descent is developed, and is tested by normalization of extrinsic features in complex fractionated atrial electrograms (CFAE).

**Method:**

Equations for normalization of x-axis and y-axis shift and scale are first derived. The algorithm is implemented for real-time analysis of CFAE acquired during atrial fibrillation (AF). Data was acquired at a 977 Hz sampling rate from 10 paroxysmal and 10 persistent AF patients undergoing clinical electrophysiologic study and catheter ablation therapy. Over 24 trials, normalization characteristics using the new algorithm with four weights were compared to the Widrow-Hoff LMS algorithm with four tapped delays. The time for convergence, and the mean squared error (MSE) after convergence, were compared. The new LMS algorithm was also applied to lead aVF of the electrocardiogram in one patient with longstanding persistent AF, to enhance the F wave and to monitor extrinsic changes in signal shape. The average waveform over a 25 s interval was used as a prototypical reference signal for matching with the aVF lead.

**Results:**

Based on the derivation equations, the y-shift and y-scale adjustments of the new LMS algorithm were shown to be equivalent to the scalar form of the Widrow-Hoff LMS algorithm. For x-shift and x-scale adjustments, rather than implementing a long tapped delay as in Widrow-Hoff LMS, the new method uses only two weights. After convergence, the MSE for matching paroxysmal CFAE averaged 0.46 ± 0.49μV^2^/sample for the new LMS algorithm versus 0.72 ± 0.35μV^2^/sample for Widrow-Hoff LMS. The MSE for matching persistent CFAE averaged 0.55 ± 0.95μV^2^/sample for the new LMS algorithm versus 0.62 ± 0.55μV^2^/sample for Widrow-Hoff LMS. There were no significant differences in estimation error for paroxysmal versus persistent data. From all trials, the mean convergence time was approximately 1 second for both algorithms. The new LMS algorithm was useful to enhance the electrocardiogram F wave by subtraction of an adaptively weighted prototypical reference signal from the aVF lead. The extrinsic weighting over 25 s demonstrated that time-varying functions such as patient respiration could be identified and monitored.

**Conclusions:**

A new LMS algorithm was derived and used for normalization of the extrinsic features in CFAE and for electrocardiogram monitoring. The weighting at convergence provides an estimate of the degree of similarity between two signals in terms of x-axis and y-axis shift and scale. The algorithm is computationally efficient with low estimation error. Based on the results, proposed applications include monitoring of extrinsic and intrinsic features of repetitive patterns in CFAE, enhancement of the electrocardiogram F wave and monitoring of time-varying signal properties, and to quantitatively characterize mechanistic differences in paroxysmal versus persistent AF.

## Introduction

In previous work it was shown that any pattern can be described based upon its intrinsic versus extrinsic features [1,2]. The extrinsic features are those that can be normalized in a signal space. The intrinsic component is the final shape of the signal following normalization. Thus intrinsic features are those measured after normalization of the space. Normalization is essential for determining the similarity of the intrinsic component between two different signals, and the normalized weighting is a measure of extrinsic differences. Testing the similarity between signals in this way is a form of pattern recognition [3,4]. Similarity measurements are also useful for noise cancellation when one signals acts as a noise reference with respect to another [5-7]. The intrinsic signal component may become apparent by averaging [1,2]. Yet, patterns lacking stationarity of the mean cannot be characterized in this way. Therefore, when signal statistics are time-varying, as is often the case, it is desirable to use adaptive analysis methods.

Least mean squares (LMS) algorithms adapt the mean squared error of a reference signal with respect to a desired signal [5,6,8]. The error is estimated at the current time, and the error gradient is approximated by the gradient from a single sample. Adaptation occurs by iterating toward the minimum of the error function. A drawback is that if local minima exist along the path, convergence to the global minimum can only occur if the weight update steps are sufficiently large to shift the convergence path out of the concavity of the local minima. In this study an LMS algorithm is derived and implemented for real-time normalization of extrinsic signal features. For simplicity, initial conditions are set to enable convergence to global rather than local minima. The algorithm is applied to complex fractionated atrial electrograms (CFAE), which are electrograms with multiple continuous deflections or cycle length <120 milliseconds [9] acquired from the heart surface that result from passage of the electrical activation wavefront. CFAE were selected in part because the presence of randomness provides a rigorous test of the capacity to rapidly and accurately estimate signal differences. Furthermore, if the acquired signals can be successfully characterized prior to catheter ablation [9], it would be possible to glean knowledge concerning abnormal conduction caused by ischemia, infarction, or the presence of fibrosis, which can be assistive to guide the catheter toward optimal ablation sites.

## Method

### Clinical data acquisition

Electrograms were recorded from 20 patients referred to the Columbia University Medical Center cardiac electrophysiology (EP) laboratory for catheter ablation of AF. These were obtained prospectively as approved by the Internal Review Board at Columbia University Medical Center, but analyzed retrospectively after the catheter ablation procedures were completed using standard clinical protocols. Ten patients had documented clinical paroxysmal AF, with a normal sinus rhythm as their baseline rhythm in the electrophysiology laboratory. Atrial fibrillation was induced by burst pacing from the coronary sinus or from the right atrial lateral wall, and the arrhythmia persisted for at least 10 minutes for those signals that were included in retrospective analysis. Ten other patients had longstanding persistent AF, and had been in AF without interruption for at least several months prior to the catheter mapping and ablation procedure. Electrograms recorded from the distal ablation electrode during arrhythmia were filtered with a single-pole bandpass from 30-500 Hz by the acquisition system to remove baseline drift and high frequency noise, sampled at 977 Hz, and stored (CardioLab, GE Healthcare, Waukesha, WI).

Only signals identified as CFAEs by two cardiac electrophysiologists were included in the retrospective analysis. CFAE recordings of at least 20 seconds in duration were obtained from two sites outside the ostia of each of the four pulmonary veins. Similar recordings were obtained at two sites in the mid-posterior left atrial free wall, and from the anterior ridge at the base of the left atrial appendage. A total of 12 paroxysmal and 12 persistent CFAE sequences, selected at random, were used for measurement and statistical comparison.

### Definition of intrinsic versus extrinsic features

**Figure 1:**
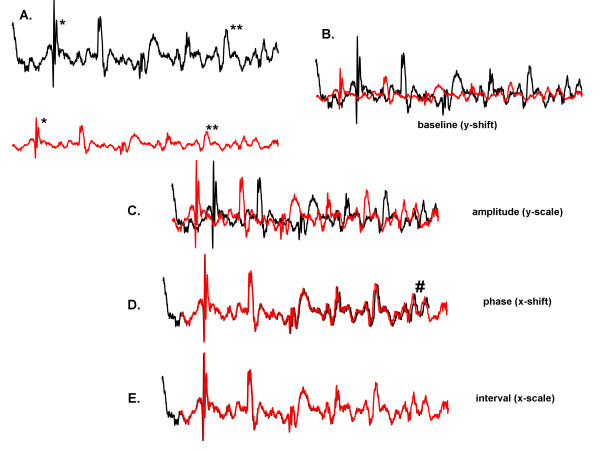
x- and y-axis scale and shift to normalize the extrinsic features of CFAE.

Consider two signals that are identical except for x-axis and y-axis shift and scale (Figure 1A). Similar deflections occur on each trace (see for example at * and **). As a first approximation toward normalizing these signals, their baselines, or degree of y-axis shift, can be globally adjusted (Figure 1B). Further normalization can be done by increasing the y-axis scale, or gain, of the red trace to better match it to the black trace (Figure 1C). If the x-axis shift, or phase lag, is adjusted, the two traces can be made to approximately align (Figure 1D).Yet there is still some misalignment due to differences in the degree of expansion along the x-axis, as noted by the # symbol in Figure 1D. If the x-axis scale is correctly adjusted, these traces perfectly match over the interval during which they both occur (Figure 1E). The signals are then normalized. The difference in extrinsic shape is then zero, and these particular signals have the same intrinsic shape.

**Figure 2:**
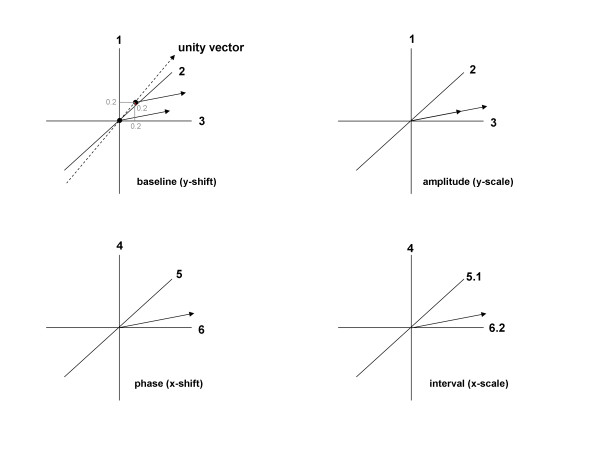
Differences in extrinsic features of signal space, shown as graphs.

Changes to the signal space that occur when extrinsic features are normalized are shown in a graphical representation in Figure 2. Three dimensions of the N-dimensional signal space are shown. In all panels, the vector direction is the intrinsic shape of the signal and it is unchanging. Baseline shift is represented by the position of the tail of the signal vector along the unity vector (Figure 2A). To remove the baseline level, the signal vector is moved from any nonzero location along the unity vector, for example at (0.2, 0.2, 0.2 …) that is, a baseline level of 0.2 units, to the origin at (0, 0, 0, …) which has a baseline level of zero. The signal amplitude is represented by the signal vector magnitude (Figure 2B). To adjust signal amplitude, the vector magnitude is adjusted. To adjust the phase lag, the signal space is rotated, or equivalently, the axes are renumbered. For example to adjust by +3 sample points, the axes are renumbered as shown in Figure 2C. This is done for all N axes, with wraparound from axis N to axis 1. Finally, to adjust x-axis scale, the sampling interval i.e., the time interval between sample points, is adjusted, for example as shown in Figure 2D. Here the sampling interval, originally 1 unit (such as 1 millisecond) is adjusted to 1.1 units. Based on these parameters, the signal space can be normalized so that extrinsic relationships between signals are removed and only intrinsic relationships remain.

By developing an LMS algorithm to normalize CFAE signals for x-axis and y-axis shift and scale, differences which are physiologically relevant can become apparent. For example, the baseline level in electrogram signals may be related to injury current gradients present at border zones where ischemia is present [10]. The amplitude is in part a result of the degree of tissue activating at any one time, although if bipolar recording electrodes are used this will also depend on the orientation and spacing of bipoles [11]. The phase lag is a direct result of the delay in arrival of the electrical activation wavefront at the recording site. The time duration or x-axis scale depends on conduction velocity, and will increase with a decrease in velocity [12]. A method to measure and normalize extrinsic signal features is described as follows.

### Description of the new LMS algorithm

We suppose a matched filter that consists of an unknown input or desired signal d and an adaptively weighted known or reference signal x. The method of steepest descent can be used to update the weight w [5,6]:

(1) $${\text{w}}_{\text{k}+1}={\text{w}}_{\text{k}}-\mu \nabla_{\text{k}}$$

where μ is the convergence coefficient, k is the discrete time, and the gradient is given by [13]:

(2) $$\nabla_{\text{k}}=\partial \text{E}\left[{\epsilon_{\text{k}}}^{2}\right]/\partial {\text{w}}_{\text{k}}=\partial \xi_{\text{k}}/\partial {\text{w}}_{\text{k}}$$

with ∂ being the partial derivative with respect to the particular weight parameter w, E the expectation operator, ϵ_k_ is the error, and ξ_k_ is the mean squared error (MSE) at discrete time k. The gradient defines the error surface (performance surface). The optimal weight vector (Wiener-Hopf equation), which is derived from the gradient vector for all parameters, requires matrix inversion for computation. However, a difference equation can be written to estimate the gradient for any parameter [8,14]:

(3) $$\nabla_{\text{k}}\approx \Delta {\epsilon_{\text{k}}}^{2}/\Delta {\text{w}}_{\text{k}}=\left[{\left({\epsilon_{\text{k}}}^{+}\right)}^{2}-{\left({\epsilon_{\text{k}}}^{-}\right)}^{2}\right]/\Delta {\text{w}}_{\text{k}}$$

where ϵ_k_^+^ and ϵ_k_^-^ are errors with finite differences, described as [15-17]:

(4) $$\epsilon_{\text{k}}={\text{d}}_{\text{k}}-{\text{y}}_{\text{k}}$$

(5) $${\epsilon_{\text{k}}}^{+}={\text{d}}_{\text{k}}-{{\text{y}}_{\text{k}}}^{+}$$

(6) $${\epsilon_{\text{k}}}^{-}={\text{d}}_{\text{k}}-{{\text{y}}_{\text{k}}}^{-}$$

and d_k_ is the signal to be emulated, with estimates given by:

(7) $${\text{y}}_{\text{k}}=\text{f}\left({\text{w}}_{\text{k}},{\text{x}}_{\text{k}}\right)$$

(8) $${{\text{y}}_{\text{k}}}^{+}=\text{f}\left({\text{w}}_{\text{k}}+\omega ,{\text{x}}_{\text{k}}\right)$$

(9) $${{\text{y}}_{\text{k}}}^{-}=\text{f}\left({\text{w}}_{\text{k}}–\omega ,{\text{x}}_{\text{k}}\right)$$

The reference signal is x_k_, while ω is the mesh spacing used for gradient estimation. Substituting Eq. 4b and 4c into Eq. 3:

(10) $$\nabla_{\text{k}}\approx \left[{\left({\epsilon_{\text{k}}}^{+}\right)}^{2}-{\left({\epsilon_{\text{k}}}^{-}\right)}^{2}\right]/\Delta {\text{w}}_{\text{k}}$$

(11) $$=\left[{\left({\text{d}}_{\text{k}}-{{\text{y}}_{\text{k}}}^{+}\right)}^{2}-{\left({\text{d}}_{\text{k}}-{{\text{y}}_{\text{k}}}^{-}\right)}^{2}\right]/\Delta {\text{w}}_{\text{k}}$$

(12) $$=\left[{{\text{d}}_{\text{k}}}^{2}-{2\text{d}}_{\text{k}}{{\text{y}}_{\text{k}}}^{+}+{\left({{\text{y}}_{\text{k}}}^{+}\right)}^{2}-{{\text{d}}_{\text{k}}}^{2}+{2\text{d}}_{\text{k}}{{\text{y}}_{\text{k}}}^{-}-{\left({{\text{y}}_{\text{k}}}^{-}\right)}^{2}\right]/\Delta {\text{w}}_{\text{k}}$$

(13) $$=\left[-{2\text{d}}_{\text{k}}\left({{\text{y}}_{\text{k}}}^{+}-{{\text{y}}_{\text{k}}}^{-}\right)+\left({{\text{y}}_{\text{k}}}^{+}+{{\text{y}}_{\text{k}}}^{-}\right)\left({{\text{y}}_{\text{k}}}^{+}-{{\text{y}}_{\text{k}}}^{-}\right)\right]/\Delta {\text{w}}_{\text{k}}$$

Supposing for finite differences, that:

(14) $${{\text{y}}_{\text{k}}}^{+}+{{\text{y}}_{\text{k}}}^{-}\approx {2\text{y}}_{\text{k}}$$

then [15-17]:

(15) $$\nabla_{\text{k}}\approx \left[-{2\text{d}}_{\text{k}}\left({{\text{y}}_{\text{k}}}^{+}-{{\text{y}}_{\text{k}}}^{-}\right)+{2\text{y}}_{\text{k}}\left({{\text{y}}_{\text{k}}}^{+}-{{\text{y}}_{\text{k}}}^{-}\right)\right]/\Delta {\text{w}}_{\text{k}}\;$$

(16) $$=-2\epsilon_{\text{k}}\left({{\text{y}}_{\text{k}}}^{+}-{{\text{y}}_{\text{k}}}^{-}\right)/2\omega$$

where ω is the mesh size as in Eq. 5, and substitution was done using Eq. 4a. To adjust the step size of the weight update by the method of steepest descent, a convergence coefficient can be added:

(17) $$\mu \nabla_{\text{k}}=-2\mu \epsilon_{\text{k}}\left({{\text{y}}_{\text{k}}}^{+}-{{\text{y}}_{\text{k}}}^{-}\right)/2\omega$$

Eq. 9 can be used to update a set of normalization weights to shift and scale the signal along the x and y axes. For y-axis scale:

(18) $$\mu_{\text{g}}\nabla_{\text{gk}}=-2\mu_{\text{g}}\epsilon_{\text{k}}\left[\left(\text{g}+\gamma \right){\text{x}}_{\text{k}}-\left(\text{g}-\gamma \right){\text{x}}_{\text{k}}\right]/2\gamma$$

(19) $$=-2\mu_{\text{g}}\epsilon_{\text{k}}2\gamma {\text{x}}_{\text{k}}/2\gamma$$

(20) $$=-2\mu_{\text{g}}\epsilon_{\text{k}}{\text{x}}_{\text{k}}$$

where ‘g’ is the signal gain and γ is the mesh size. Eq. 10 is the Widrow-Hoff LMS algorithm in scalar form [5,6]. For y-axis shift, the gradient is:

(21) $$\mu_{\text{b}}\nabla_{\text{bk}}=-2\mu_{\text{b}}\epsilon_{\text{k}}\left[\text{g}\left({\text{x}}_{\text{k}}+\text{b}+\beta \right)-\text{g}\left({\text{x}}_{\text{k}}+\text{b}-\beta \right)\right]/2\beta$$

(22) $$=-2\mu_{\text{b}}\epsilon_{\text{k}}2\beta /2\beta$$

(23) $$=-2\mu_{\text{b}}\epsilon_{\text{k}}$$

where ‘b’ is the DC bias and β is the mesh size. Eq. 11 is the Widrow-Hoff LMS update of the DC or average level [5,6]. For x-axis shift (phase lag):

(24) $$\mu_{\text{p}}\nabla_{\text{pk}}=-2\mu_{\text{p}}\epsilon_{\text{k}}\left[\text{g}\left({\text{x}}_{\text{k}+\text{p}+\phi }+\text{b}\right)-\text{g}\left({\text{x}}_{\text{k}+\text{p}-\phi }+\text{b}\right)\right]$$

(25) $$=-2\mu_{\text{p}}\epsilon_{\text{k}}\text{g}\left({\text{x}}_{\text{k}+\text{p}+\phi }-{\text{x}}_{\text{k}+\text{p}-\phi }\right)$$

where ‘p’ denotes the phase shift, ϕ is the mesh size, and for simplicity the constant term 1/2ϕ is included in μ_p_. For x-axis scale (interval weighting):

(26) $$\mu_{\text{a}}\nabla_{\text{ak}}=-2\mu_{\text{a}}\epsilon_{\text{k}}\left[\text{g}\left({\text{x}}_{(\text{a}+\alpha )\text{k}}+\text{b}\right)-\text{g}\left({\text{x}}_{(\text{a}-\alpha )\text{k}}+\text{b}\right)\right]$$

(27) $$=-2\mu_{\text{a}}\epsilon_{\text{k}}\text{g}\left({\text{x}}_{(\text{a}+\alpha )\text{k}}-{\text{x}}_{(\text{a}-\alpha )\text{k}}\right)$$

where ‘a’ denotes x-axial scale, α is the mesh size, and again for simplicity the constant term 1/2α is included in μ_a_. Because the time index becomes a real number when adjusting for x-axis shift and scale, interpolation between discrete sample points is necessary. Eqs. 1013 can be used in tandem to update the values of x-axis and y-axis scale and shift, once per discrete time epoch k. Accordingly, the equations for updating x-axis shift and scale are rewritten as:

(28) $$\mu_{\text{p}}\nabla_{\text{pk}}=-2\mu_{\text{p}}\epsilon_{\text{k}}\text{g}\left({\text{x}}_{\text{a}(\text{k}+\text{p}+\phi )}-{\text{x}}_{\text{a}(\text{k}+\text{p}-\phi )}\right)$$

(29) $$\mu_{\text{a}}\nabla_{\text{ak}}=-2\mu_{\text{a}}\epsilon_{\text{k}}\text{g}\left({\text{x}}_{(\text{a}+\alpha )\left(\text{k}+\text{p}\right)}-{\text{x}}_{(\text{a}-\alpha )\left(\text{k}+\text{p}\right)}\right)$$

and:

(30) $$\epsilon_{\text{k}}={\text{d}}_{\text{k}}-{\text{y}}_{\text{k}}$$

(31) $$={\text{d}}_{\text{k}}-{\text{g x}}_{\text{a}\left(\text{k}+\text{p}\right)}+\text{b}$$

### Comparison of new and widrow-Hoff LMS algorithms

The performance of the new and Widrow-Hoff LMS algorithms was compared using CFAE, signals with significant randomly-varying deflections as well as repetitive components [18,19]. Equations 1016 were used to match CFAE signals of length = 8,192 discrete sample points (8.4 s), which are the desired signals d_k_ for all k, with versions of the same signal that were shifted and scaled along the x and y axes by a known amount, which are the reference signals x_k_ for all k. Thus upon convergence, the weights will be inverse values to the x-axis and y-axis shift and scale that was applied to form each reference x_k_. We used CFAE that were initially 16,384 sample points long for this application, because the x-axis shift and scale applied to form x_k_ could require values of k > 8192 in the original signal. In twelve trials, weighting initialization for reference signal x of dimension 8,192, was based upon random adjustment of x-axis and y-axis shift and scale for each trial, within limits of the concavity of the global minimum along the four-dimensional performance surface. To determine efficacy, 12 paroxysmal and 12 persistent CFAE recordings were used (24 trials in all).

Although the mean-squared error surface of y-axis shift and scale forms a single concavity along the performance surface, the mean-squared error surface of x-axis shift and scale can have multiple concavities, i.e. local minima, due to the presence of multiple signal deflections. If during a given trial, the weighting did not converge at the global minimum error value due to arrival at a local minimum, the trial was excluded from analysis, and was redone with another set of initial weight values. The convergence coefficient magnitudes, the same for all trials, were selected manually for fastest convergence, without significant response, after convergence, of the weighting to individual signal deflections, so that the weighting foci upon convergence to the bottom of weight bowl had small footprint. The computer code to implement these equations is given in the Appendix.

**Figure 3:**
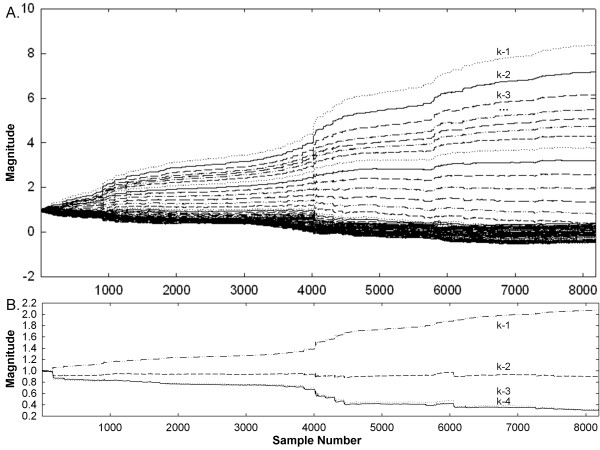
Using the Widrow-Hoff LMS algorithm for adaptive weight update over 8192 sample points A. tapped delay line of 100. B. tapped delay line of 4.

For comparison of estimation error and convergence time, the Widrow-Hoff LMS algorithm [5,6,8] was implemented with the same desired signal d and the same reference input x adjusted for x- and y- shift and scale. Initially a tapped delay line of length 100 was used for Widrow-Hoff, so that variations in x-axis scale could be better accounted for as d_k_ became increasingly out of phase with x_k_ when a ≠ 1 (Eqs. 15 and 16). However, it was observed that the relationship between many of the individual weights changed little even for p ≠ 0 and a ≠ 1. This is illustrated in Figure 3A. When 100 delays are used, taps in recent time (k = −1, -2, -3 …) are large but those furthest in time (k = … -91 to −100) remain near zero (bottom part of panel). Thus much of the weighting was relatively unaffected by phase lag differences between d and x. When only four taps were used (Figure 3B) there is more contribution from each weight (values are nonzero) and the lesser number of taps increases the computational efficiency. Thus for comparison, Widrow-Hoff with four taps was compared with the new LMS algorithm with four weights. For Widrow-Hoff, the convergence coefficient value was also selected for fast convergence without significant response to individual signal deflections following convergence (small foci at bottom of weight bowl).

The new LMS algorithm was initialized with weights g = 1, b = 0, p = 0, and a = 1 in all trials, i.e., supposing that there are no extrinsic differences between signals. The Widrow-Hoff LMS algorithm was initialized with weights for all four taps = 1 in all trials. The weights were considered to be converged following the cessation of sharp trends of weight change. The error after convergence was then calculated for both LMS algorithms as:

(32) $$\text{error}=\Sigma_{\text{k}}{\left({\text{d}}_{\text{k}}-{\text{y}}_{\text{k}}\right)}^{2}/2000.\text{k}=\text{j}+1,\text{j}+2000$$

where j is the start of the interval just after convergence for that algorithm which took longer to converge, y_k_ is given by Eq. 16 for the new LMS algorithm, and for the Widrow-Hoff LMS algorithm:

(33) $${\text{y}}_{\text{k}}={\underset{̅}{\text{w}}}^{\text{T}}\cdot \text{x}\left(\text{k}:\text{k}+3\right)$$

where ‘·’ denotes the inner product and w is of dimension 4. The mean and standard deviation in the error (Eq. 17) and the time for convergence were tabulated for each trial.

Significant differences in the means between the new and Widrow-Hoff LMS were determined using the unpaired *t*-test, with p < 0.05 indicating significance (SigmaPlot 2004version 9.01, Systat Software, Chicago, IL, and MedCalc 2011 version 9.5, MedCalc Software, Mariakerke Belgium).

### Application to the electrocardiogram

As proof of concept to show the utility of the new LMS algorithm with other types of AF signals, it was applied to lead aVF of the electrocardiogram in a patient with longstanding persistent AF. Due to the irregularity of atrial activation during AF, ventricular activation is also often irregular. Over a 25 s interval, a prototypical waveform was matched with the actual aVF lead. The prototypical waveform was constructed as the average of all QRS complexes over the 25 s sequence. The prototypical waveform consisted of ±400 sample points centered on the R wave peak (820 ms duration). A synthetic electrocardiogram was constructed by inserting this prototypical waveform at each instance of and aligned with the R wave peak of the actual aVF lead along a 977samples/s × 25 s sample point interval. Where no instance of the prototypical waveform occurred (cardiac cycle > 820 ms), the sequence was set to zero. Where two instances of the prototypical waveform overlapped (cardiac cycle < 820 ms), they were summed. The resultant synthetic electrocardiogram served as x, while the original electrocardiogram was d. The weighting of x was initialized at random to values of:

(34) $$\left(\text{b},\text{g},\text{p},\text{a}\right)=\left(-0.2,1.3,-1.,1.02\right)$$

to form the estimate y. The electrocardiogram statistical characteristics differed from that of surface electrograms, and different convergence coefficients were used:

(35) $$\left(\mu_{\text{b}},\mu_{\text{p}},\mu_{\text{g}},\mu_{\text{a}}\right)=\left(0.0005,0.5,0.02,0.004\right)$$

Signals d, y, and ϵ were graphed prior to and after convergence. Extrinsic weights b, g, p, and a were graphed for the entire 25 s sequence.

## Results

**Figure 4:**
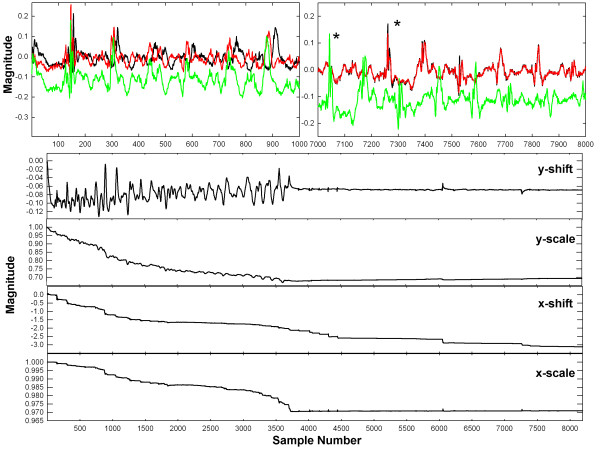
**Adaptive update using the new algorithm (Trial 6 in Table **1**).** The top panels show the desired signal (black), reference signal (green) and estimated signal (red). The lower panels show the adjustment of weights for x- and y-axis shift and scale.

In Figure 4 is shown an example of the adaptive update using the new algorithm with a CFAE, trial 6, paroxysmal AF (see Table 1). The top panels show the desired signal (black), the reference (green) and the estimated signal (red). At top left is shown the onset of adaptation, sample points 1–1000. There is initially poor overlap of estimate signal y (red) to desired signal d (black). Based on the reference signal x (green) there is substantial x-axis scaling, as the deflections increasingly misalign from left to right in the panel. However, during the interval from 7000–8000 sample points (right-hand panel) convergence has occurred and there is overlap between desired and estimated signals (black and red, respectively). The reference signal during this interval (green) is highly out of phase with the desired signal due to its nonunity weighting in x-scale. For example the first large deflection in the reference signal (green) occurs at approximately 7050, and in the desired and estimated signals (black and red traces, respectively) this deflection occurs at ~7250 (noted by *). The lower panels in Figure 4 show the weight adaptation. Convergence occurs at approximately sample point 3742, in the sense that rapid drifts in weight values cease to occur. The value of each weight at convergence is approximately the inverse of the corresponding value in Table 1, trial 6, so that the desired and estimated signals match.

**Table 1:** Trials for New LMS Algorithm – Paroxysmal AF

**Trial**	**y-shf**	**y-sca**	**x-shf**	**x-sca**	**MN (μV**^**2**^**)**	**SD (μV**^**2**^**)**	**Converge**
1	0.2	1.5	4	0.99	0.048	0.473	64
2	0.2	1.5	2	0.99	0.411	2.363	109
3	0.2	2.0	0	1.04	1.386	9.733	87
4	−0.2	2.0	0	1.04	1.248	6.198	118
5	0.6	1.2	3	0.99	1.007	2.989	56
6	0.1	1.4	3	1.03	0.112	1.125	3742
7	0.1	1.4	2	0.985	0.078	0.0822	145
8	0.05	0.95	1	0.989	0.232	2.743	7
9	−0.5	1.5	8	1.05	0.567	4.721	1020
10	−0.5	1.5	4	1.07	0.303	0.898	1801
11	0.0	1.0	0	1.00	0.000	0.000	0
12	0.2	1.0	0	1.01	0.159	0.627	501
Mean	--	--	--	--	0.46 ± 0.49	2.66 ± 2.94	638 ± 1117

MN = mean, SD = standard deviation, shf = shift, sca = scale, converge = number of discrete time points for convergence to occur, μV = microvolts.

**Figure 5:**
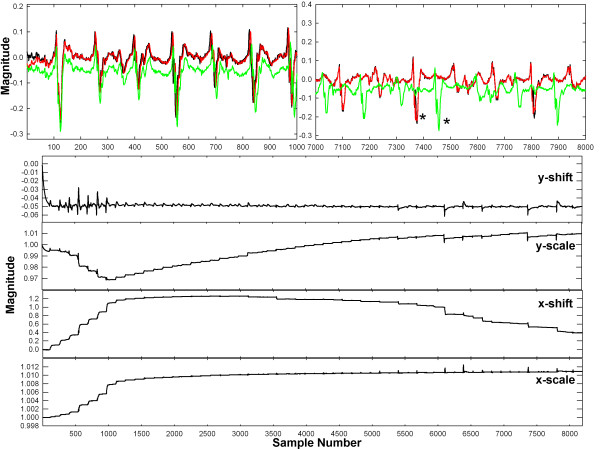
**Adaptive update using the new algorithm using different parameters (Trial 8 in Table **2**).** The top panels show the desired signal (black), reference signal (green) and estimated signal (red). The lower panels show the adjustment of weights for x- and y-axis shift and scale.

In Figure 5 is shown an example of the adaptive update using the new algorithm, trial 8, persistent AF (see Table 2). As in Figure 4, the top panels show the desired signal (black), reference (green) and estimate signal (red). The desired and reference signals again become out of phase due to a difference in x-axis scale, but the x-scaling is in the opposite direction as compared with Figure 4 (see for example peaks at *). The lower panels show the adjustment of weights for x- and y-axis shift and scale. There is mostly convergence around sample point 1100, as is evident also in the top left panel. Thus even with quite different x- and y-axial shift and scale, adaptive adjustment and normalization of the reference signal toward the shape of the desired signal occurs. The value of the shift and scale weights upon convergence show the difference in extrinsic features between desired and reference signals.

**Table 2:** Trials for New LMS Algorithm – Persistent AF

**Trial**	**y-shf**	**y-sca**	**x-shf**	**x-sca**	**MN (μV**^**2**^**)**	**SD (μV**^**2**^**)**	**Converge**
1	0.2	1.5	4	0.99	0.155	0.880	1559
2	0.2	1.5	2	0.99	0.104	0.393	2201
3	0.2	2.0	0	1.04	2.500	15.979	84
4	−0.2	2.0	0	1.04	0.100	0.606	282
5	0.6	1.2	3	0.99	0.511	1.340	125
6	0.1	1.4	3	1.03	0.069	0.398	955
7	0.1	1.4	2	0.985	2.614	6.225	50
8	0.05	0.95	1	0.989	0.067	0.232	1139
9	−0.5	1.5	8	1.05	0.197	0.699	6002
10	−0.5	1.5	4	1.07	0.099	0.294	6134
11	0.0	1.0	0	1.00	0.000	0.000	0
12	0.2	1.0	0	1.01	0.198	1.251	190
Mean	--	--	--	--	0.55 ± 0.95	2.36 ± 4.60	1560 ± 2217

MN = mean, SD = standard deviation, shf = shift, sca = scale, converge = number of discrete time points for convergence to occur, μV = microvolts.

**Figure 6:**
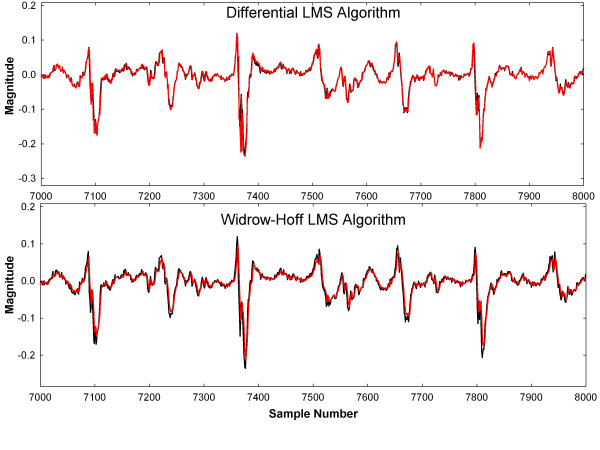
**Comparison of new versus Widrow-Hoff LMS algorithm (Trial 1 in Table **2**).**

A comparison of new versus Widrow-Hoff LMS algorithm during Trial 1, persistent AF, is shown in Figure 6. Shown after convergence, there is nearly exact overlap of desired and estimated signals using the new LMS algorithm (top panel). The Widrow-Hoff LMS algorithm matches desired and estimated signals to a slightly lesser degree - there is some misalignment in phase and amplitude. This is likely due to the need of a delay line with multiple taps to estimate changes in x-shift and x-scale.

The overall findings of this study are summarized in Tables 1, 2, 3 and 4. In Table 2 are depicted the results for the new LMS algorithm, persistent AF. Shown are the trial number, the parameter values for initialization of each trial, the mean and standard deviation in error after convergence, and the number of sample points necessary for approximate convergence. The y-shift (baseline level) of the reference takes on both positive and negative values in the individual trials. The y-scale (signal amplitude), is varied from 0.95× to 2×. The x-shift (phase lag), is varied by up to 8 units. Finally, the x-scale (time interval), is both >1 (signal contraction) and <1 (signal expansion). The error is similar for all trials with a mean of 0.55 ± 0.95 μV^2^/sample point. The mean number of sample points for convergence is 1560 ± 2217. Convergence is approximately proportional to the absolute difference between no change in x-scale (a = 1) and the value of x-scale that was actually used to initialize the reference signal (5^th^ column from left).

**Table 3:** Trials for Widrow-Hoff LMS Algorithm – Paroxysmal AF

**Trial**	**y-shf**	**y-sca**	**x-shf**	**x-sca**	**MN (**μ**V**^**2**^**)**	**SD (**μ**V**^**2**^**)**	**Converge**
1	0.2	1.5	4	0.99	0.196	0.881	23
2	0.2	1.5	2	0.99	0.925	4.239	15
3	0.2	2.0	0	1.04	1.244	9.361	114
4	−0.2	2.0	0	1.04	1.068	5.877	14
5	0.6	1.2	3	0.99	0.461	2.137	28
6	0.1	1.4	3	1.03	0.625	3.198	1971
7	0.1	1.4	2	0.985	0.232	1.395	10
8	0.05	0.95	1	0.989	0.849	5.466	5
9	−0.5	1.5	8	1.05	0.903	6.025	1386
10	−0.5	1.5	4	1.07	0.740	2.225	83
11	0.0	1.0	0	1.00	0.318	1.319	96
12	0.2	1.0	0	1.01	1.022	2.835	93
Mean	--	--	--	--	0.72 ± 0.35	3.75 ± 2.52	320 ± 648

MN = mean, SD = standard deviation, shf = shift, sca = scale, converge = number of discrete time points for convergence to occur, μV = microvolts.

**Table 4:** Trials for Widrow-Hoff LMS Algorithm – Persistent AF

**Trial**	**y-shf**	**y-sca**	**x-shf**	**x-sca**	**MN (μV**^**2**^**)**	**SD (μV**^**2**^**)**	**Converge**
1	0.2	1.5	4	0.99	0.567	2.021	156
2	0.2	1.5	2	0.99	0.499	1.394	434
3	0.2	2.0	0	1.04	2.256	12.937	12
4	−0.2	2.0	0	1.04	0.380	1.462	820
5	0.6	1.2	3	0.99	0.312	0.824	14
6	0.1	1.4	3	1.03	0.488	1.930	1020
7	0.1	1.4	2	0.985	0.947	9.558	18
8	0.05	0.95	1	0.989	0.665	2.316	10
9	−0.5	1.5	8	1.05	0.267	0.583	2204
10	−0.5	1.5	4	1.07	0.146	0.432	589
11	0.0	1.0	0	1.00	0.417	1.120	10
12	0.2	1.0	0	1.01	0.501	2.160	207
Mean	--	--	--	--	0.62 ± 0.55	3.06 ± 3.94	458 ± 651

MN = mean, SD = standard deviation, shf = shift, sca = scale, converge = number of discrete time points for convergence to occur, μV = microvolts.

Similar results were obtained using the same initialization parameters for paroxysmal CFAE data (Tables 1 and 3). For the new LMS algorithm, the mean error was 0.46 ± 0.49 μV^2^/sample point (Table 1) as compared with 0.72 ± 0.35 μV^2^/sample point for the Widrow-Hoff algorithm (Table 3). The respective convergence times for paroxysmal AF data (Tables 1 and 3) are somewhat shorter than for persistent data (Tables 2 and 4).

The results for the Widrow-Hoff LMS algorithm, persistent AF, are shown in Table 4. The same weight values were used to initialize the reference signals (left columns). The mean error value is 0.62 ± 0.55 μV^2^/sample point. The time for convergence is sometimes very short, at other times it is long, with a mean of 458 ± 651 sample points.

For both paroxysmal and persistent AF data, the modeling of the desired signal with the reference using extrinsic shape factors was more accurate using the new LMS algorithm, though this difference did not reach significance with N = 12.

### Electrocardiogram result

**Figure 7:**
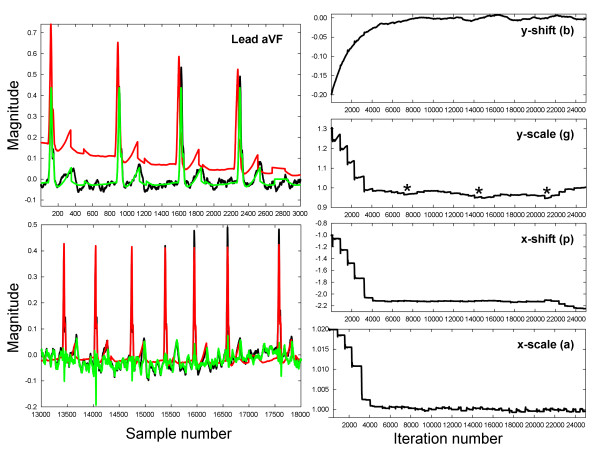
**Application of the LMS algorithm to lead aVF of the electrocardiogram in a persistent AF patient.** Left panels: the signals used for analysis. Right panels – the extrinsic weighting over a 25 s interval.

The result of the proof of concept exercise is shown in Figure 7. In the left panels are the electrocardiogram, lead aVF, during longstanding persistent atrial fibrillation. The top panel shows initial conditions leading to convergence. The black trace is the actual aVF lead, signal d. The green trace is the repeating prototypical trace, signal x. The red trace is y, the weighted manifestation of x. After convergence (bottom left panel) there is mostly overlap of d (black) with y (red). In the bottom left panel, the green trace now shows ϵ = d – y. The R wave and much of the QRS complex is eliminated. What remains is mostly the F wave (atrial signal). At right are the traces over ~25 s (each iteration number corresponds to a sample point of data, taken at 977 Hz). Convergence occurs after ~4000 iterations. The staircase shape prior to convergence in weights g, p, and reflects incidences of new cardiac cycles. The weights converge to approximately their expected values of:

(36) $$\left(\text{b},\text{g},\text{p},\text{a}\right)=\left(0,1,0,1\right)$$

Following convergence there are still slight changes in the weighting. For example, it is apparent that patient respiration is evident in the g weighting, as noted by *, although this should be verified in a prospective study.

## Discussion

### Summary

In this study a new LMS algorithm was derived for characterization of extrinsic signal features during signal normalization. These features are the x- and y- axis shift and scale parameters. The algorithm is adaptive and the weights are updated based upon the method of steepest descent (Eq. 1). The error gradient is estimated using a finite difference approximation (Eq. 8). Once coefficients are selected for fastest convergence without following individual deflections, the algorithm is stable and can adjust to a wide range of extrinsic feature values associated with a particular reference. In the study, once convergence occurred, the desired and estimated signals overlapped, i.e. their intrinsic shape was the same. The parameter values upon convergence are quantitative descriptors of differences in extrinsic signal shape, which can be related to real physiologic parameters. More rapid convergence of both LMS algorithms occurred for paroxysmal AF data (Tables 1 and 3) as compared with persistent data (Tables 2 and 4), perhaps because a greater degree of randomness in the paroxysmal deflections resulted in sharper changes in the error function, thereby increasing the step size from one iteration to the next. The new LMS algorithm enhanced the electrocardiogram F wave from a persistent AF patient. Spectral analysis of the F wave trace could be useful to characterize global atrial electrophysiologic signal during AF. The likely detection of patient respiration in the extrinsic weighting over 25 s suggests that time-varying changes can be monitored over long intervals.

### New LMS algorithm – direction of weight update

**Figure 8:**
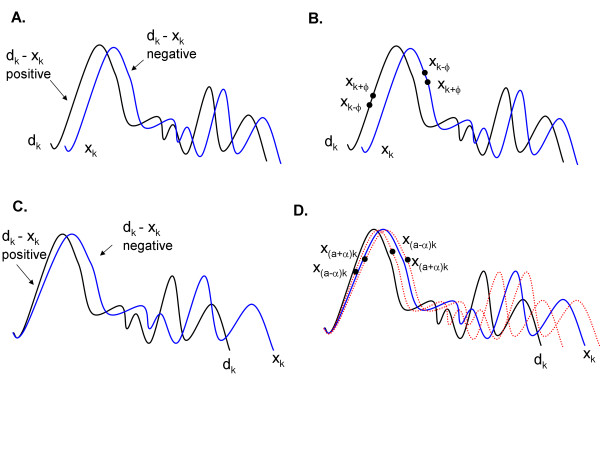
**Illustration of the mechanism by which the direction of weight update is determined for best match of weighted reference signal x with desired signal d.**

Consider how the normalization of x-axis shift is accomplished by the new LMS algorithm. To show the phase weight update direction, the relevant terms from Eqs. 14 and 16 are:

(37) $$\epsilon_{\text{k}}\left({\text{x}}_{\text{a}(\text{k}+\text{p}+\phi )}-{\text{x}}_{\text{a}(\text{k}+\text{p}-\phi )}\right)$$

(38) $$=\left({\text{d}}_{\text{k}}-{\text{g x}}_{\text{a}\left(\text{k}+\text{p}\right)}+\text{b}\right)\left({\text{x}}_{\text{a}(\text{k}+\text{p}+\phi )}-{\text{x}}_{\text{a}(\text{k}+\text{p}-\phi )}\right)$$

(39) $$=\left({\text{d}}_{\text{k}}-{\text{x}}_{\text{k}}\right)\left({\text{x}}_{\text{k}+\phi }-{\text{x}}_{\text{k}-\phi }\right)$$

where for simplicity, for Eq. 19b an initialized weighting is supposed (g = 1, b = 0, p = 0, and a = 1), i.e., y_k_ = x_k_. Consider the left-hand term in parentheses Eq. 19b. The effect of this term on the weight update is illustrated in Figure 8A. When d_k_ is more positive than the weighted version of x_k_, d_k_ - y_k_ will be positive. When d_k_ is more negative than the weighted version of x_k_, d_k_ - y_k_ will be negative. The difference between k+ϕ and k-ϕ in Eq. 19b (right-hand term) is just a difference in the phase of signal x (Figure 8B). If the slope of x is positive at k, then (x_k+ϕ_ - x_k-ϕ_) will be positive, whereas if the slope is negative, (x_k+ϕ_ - x_k-ϕ_) will be negative. Based upon Figure 8A-8B, along the upgoing slope, (d_k_ - x_k_) and (x_k+ϕ_ - x_k-ϕ_) will both be positive. Thus the phase update will be negative (Eq. 14) and x_k_ will shift to the left toward d_k_. Similarly, on the downgoing slope, (d_k_ - x_k_) and (x_k+ϕ_ - x_k-ϕ_) will both be negative and again x_k_ will be shifted to the left toward better alignment with d_k_. If the weighted version of x_k_ were leading d_k_ (not shown), the product of the terms in Eq. 19 will be negative regardless of whether k is located at an upslope or downslope of the signal, and x_k_ will be shifted to the right.

For normalization of x-scale, the relevant terms from Eqs. 15 and 16 are:

(40) $$\epsilon_{\text{k}}\left({\text{x}}_{(\text{a}+\alpha )\left(\text{k}+\text{p}\right)}-{\text{x}}_{(\text{a}-\alpha )\left(\text{k}+\text{p}\right)}\right)$$

(41) $$=\left({\text{d}}_{\text{k}}-{\text{g x}}_{\text{a}\left(\text{k}+\text{p}\right)}+\text{b}\right)\left({\text{x}}_{(\text{a}+\alpha )\left(\text{k}+\text{p}\right)}-{\text{x}}_{(\text{a}-\alpha )\left(\text{k}+\text{p}\right)}\right)$$

(42) $$=\left({\text{d}}_{\text{k}}-{\text{x}}_{\text{k}}\right)\left({\text{x}}_{(1+\alpha )\text{k}}-{\text{x}}_{(1-\alpha )\text{k}}\right)$$

supposing for Eq. 20b, as in Eq. 19b, initial conditions (g = 1, b = 0, p = 0, and a = 1). As for x-shift, the direction of weight update for x-scale depends in part on the difference between d_k_ and x_k_ (Figure 8C). In this panel, the weighting of reference signal x is illustrated by a change in x-axis scale (expansion) as compared with desired signal d, but the same relationships apply as in Figure 8A. Similar to Figure 8B for x-shift, in Figure 8D are shown the effect of finite differences on x-scale (red dotted lines). Again, the difference will be positive along a deflection leading edge and negative along a deflection trailing edge. Thus the value a of x-scale will decrease whether k is on the leading or trailing edge of a deflection (shift to left) when the weighted version of x_k_ is expanded with respect to d_k_ (Figure 8D). When the weighted version of x_k_ is contracted with respect to d_k_, the value a of x-scale will increase whether k is on the leading or trailing edge of a deflection (shift to right, not shown). Figure 8C illustrates that these relationships anticipate that the overlap between desired and weighted reference signal are sufficient for correct adaptation (large deflection at left in Figure 8C). Where smaller deflections are aligned with a non-corresponding deflection on the other signal (right portion of traces), local minima will occur in the performance surface, which will delay weight convergence to the global minimum error.

### Quantitative comparison to other applications

The approach described in this study minimizes the sum of squares of evenly weighted deviations of a template to an input signal and was specifically applied to CFAE. As a first approximation, it can be supposed that the MSE weighting in each parameter has constant variance independent of the position within the data set [20]. Another approach to the use of the method of steepest descent in adaptive problems is by frequency domain filtering, which reduces the computational cost relative to the Widrow-Hoff LMS algorithm in applications where long filter lengths are required [14]. While for simplicity, the convergence coefficient values were estimated manually in our study, this process can be automated to reflect the inverse signal power [6]. The step size depends on the position of the weights along a performance surface. In prior work, fixed step size has been used with finite differences to make adaptive updates with greater stability for simulated cardiologic data [21] as well as to cancel motion artifact from the blood pressure pulse obtained by tonometry [22]. The MSE is inversely proportional to the convergence coefficient μ [23]. In our study, convergence coefficient values for both LMS algorithms were optimized manually for best step-size. Variable step-size LMS adaptive filtering may helpful to improve convergence [24,25]. In cases when eigenvalues defining the signal statistics have a large spread, a variable leaky LMS algorithm, designed to overcome the slow convergence of the standard LMS algorithm under such conditions, may also helpful [26]. Future implementations should consider transient and steady-state responses of the LMS algorithms [27].

### Clinical correlates

In this study we tested a new LMS algorithm using CFAE, which contain both random and repetitive components which differ in paroxysmal versus persistent AF [19]. CFAE from paroxysmals tend to be less repetitive and more complex as compared with persistent AF data. In previous work, linear prediction was used to detect repetitive components [19]. For real-time analysis, once components are detected by linear prediction, the new LMS algorithm could be applied to normalize the extrinsic features. An instance of the repetitive shape early in the recorded signal would be used as the template or reference (x). The length of x would equal the interval along which the repetitive pattern is detected. The template x would be matched with new instances of the repetitive pattern d by using the four weight system of the new LMS algorithm to form the weighted template y. The x-axis scale weighting will adjust the template length to meet any changes in time duration of the recurring pattern.

Upon application of this paradigm, the variation in x-axis and y-axis shift and scale itself would provide information about the character of CFAE repetitive patterns. Furthermore, the intrinsic shapes after normalization with the new LMS algorithm could be quantified using specially designed methods, such as analysis of morphology variations by cluster and principal components analysis [28] or by nonlinear dynamical measurements [18], wave similarity analysis [29], or correlation waveform analysis [30]. We would expect to see differences in intrinsic and extrinsic features not only in paroxysmal versus persistent AF data obtained at corresponding anatomical locations, but also for data from the same patient obtained at different anatomical locations such as at the ostia of the pulmonary veins versus the anterior and posterior left atrial free wall. Guidance for ablation therapy might be possible based upon the degree of temporal change in extrinsic shape of repetitive patterns in CFAE, as well as the character of their intrinsic shape.

It was also shown that the method is applicable to more deterministic signals, specifically the electrocardiogram. The F wave was enhanced in leaf aVF in a longstanding persistent AF patient, corresponding to atrial activation. Likewise, monitoring of the extrinsic weighting of the electrocardiogram over long intervals might be useful to determine the affect of antiarrhythmic drugs or the result of RF ablation. Individual electrocardiogram components such as the R wave could likewise be monitored. This could be useful for example, during administration of pharmacologic agents that specifically alter electrical conduction in the ventricles.

## Conclusions

It was found that a new LMS algorithm can converge stably and rapidly to normalize a reference CFAE x to the same space as that of a desired CFAE signal d. All four parameters of x-axis and y-axis shift and scale converged during this process when the initial weighting was within the concavity of the global minimum error. Since each weight directly normalizes a particular aspect of extrinsic shape, the new LMS algorithm tends to converge with less error as compared with a four-tap Widrow-Hoff LMS implementation. Other algorithms have been developed which might be helpful to further reduce convergence time [31]. The new LMS method is potentially useful to determine differences in physiologic parameters for CFAE recorded at many atrial sites, based on measurements of x- and y-axis shift and scale and their variation over time. It can also be used to enhance the electrocardiogram F wave and to monitor global, time-varying changes in heart function.

### Limitations

In the study a limited number of trials were used for comparison. Only 24 different CFAE were used – one for each trial. There were also 12 different initial weighting conditions used to test the adaptation of the algorithms. For more rigorous comparison, in subsequent work the number of trials and initial conditions should be increased. Furthermore, the intrinsic signal shape of desired versus reference signals should be made to differ. For the new LMS, the step size for weight update was proportional to the finite difference in estimated signal, but updates using fixed increments [21,22,32] may also converge rapidly and stably. Initialization was set so that the path along the performance surface was within the concavity of the global minimum error. Addition of a stochastic factor (random number) in Eq. 16 may be useful to overcome local minima during update of x-shift and x-scale as caused by presence of multiple signal deflections [21]. In the study it was supposed as a first approximation that for Widrow-Hoff, the delay line with 4 taps equals the unknown system impulse response; actual response should be tested for more accurate approximation [33].

## Appendix

Computer program for new LMS algorithm

parameter (m = 8192, u1 = 0.03, u2 = .08, u3 = 4., u4 = .04, v = .001)

real d(m),x(m + 500); data b, g, p, a/0., 1., 0., 1./

do 1 k = 101, 8192 + 100

z = a*(k + p); zz = int(z); xx = x(zz) + (x(zz + 1)-x(zz))*(z-zz)

z = a*(k + p + 1); zp = int(z); xp = x(zp) + (x(zp + 1)-x(zp))*(z-zp)

z = a*(k + p-1); zm = int(z); xm = x(zm) + (x(zm + 1)-x(zm))*(z-zm)

y = (a + v)*k + p; yp = int(y); xpp = x(yp) + (x(yp + 1)-x(yp))*(y-yp)

y = (a-v)*k + p; ym = int(y); xmm = x(ym) + (x(ym + 1)-x(ym))*(y-ym)

e = d(k) - (g*xx-b)

b = b-u1*e; g = g + u2*e*xx); p = p + u3*e*g*(xp-xm); a = a + u4*e*g*(xpp-xmm)

write(*,*) d(k), g*x(a*(k + p))-b, x(k), b, g, p, a

1 continue

The Fortran program (Intel Visual Fortran Compiler, ver. 9, 2005, Intel Corp., Santa Clara, CA) calculates error and update weights, and writes signals and weights to disk.

u are convergence coefficients, b, g, p, a are y- and x-axis shift & scale weights.

d = desired signal, x = reference signal, v is the differential for x-axis scale.

y, yp, ym and z, zp, zm are intermediate calculations of x-axis shift and scale, respectively.

xx is the calculation of x-axis shift and scale without finite difference.

xp and xm are differential calculations of x-axis shift.

xpp and xmm are differential calculations of x-axis scale.

k begins at 100 to prevent the possibility of a negative index in x-axis scale and shift.

x is extended to 8192 + 500 to account for positive ending x-axis scale and shift.

## Competing interests

The authors declare that they have no competing interests.

## Author contributions

EJC developed the LMS algorithm, conducted the data analysis, and wrote the manuscript. ABB, WW, and HG made helpful suggestions, provided the clinical data, and determined which recordings were complex fractionated atrial electrograms. All authors have read and approved the final manuscript.
